# Risk Communication as Government Agency Organizational Practice

**DOI:** 10.1111/risa.13302

**Published:** 2019-03-18

**Authors:** Åsa Boholm

**Keywords:** Government agencies, practitioners, regulation

## Abstract

The dynamics of organizational risk communication is an understudied topic in risk research. This article investigates how public officials at six government agencies in Sweden understand and relate to risk communication and its uses in the context of agency organizational work on policy and regulation. Qualitative interviews were used to explore the practitioners’ views on some key topics in the academic literature on risk communication. A main finding is that there is little consensus on what the goals of risk communication are; if, and how, uncertainty should be communicated; and what role is to be played by transparency in risk communication. However, the practitioners agree that dissemination (top down) to the public of robust scientific and expert knowledge is a crucial element. Dialogue and participation is used mainly with other agencies and elite stakeholders with whom agencies collaborate to implement policy goals. Dialogue with the public on issues of risk is very limited. Some implications of the findings for the practice of risk communication by government agencies are suggested.

## 1. INTRODUCTION

Risk communication is both something that practitioners do as part of “risk work” in organizational settings (Power, 2016) and a field of academic study of how risk is, or should be, communicated, including theories, concepts, methods, findings, and recommendations (Otway & Wynne, 1989; Plough & Krimsky, 1987). Risk communication as an academic field focuses on exploratory, explanatory, and normative accounts of the practice of risk communication. It investigates and evaluates communicative events, practical management, and policy regarding risk (Grabill & Simmons, 1998; Gutteling, 2015; Heath & O'Hair, 2010; McComas, 2006; Palenchar & Heath, 2007; Plough & Krimsky, 1987; Wardman, 2008).

Risk communication practice is essential to policy and regulation covering policy fields such as environment, natural resource management, the built environment, infrastructure, chemicals, food, energy, and contingency preparedness. Government agencies are responsible for communicating assessments of potential hazards and their management to affected groups, stakeholders, and the general public. As part of regulatory policy work they communicate about risk with other government agencies, nongovernmental organizations, industry, and the media. Practitioners at government agencies communicate about risk on an everyday basis as part of their duties and work practice.

Early academic risk communication studies were often explicitly intended to improve practice (Hance, Chess, & Sandman, 1988; Plough & Krimsky, 1987). Ambitious guidelines, recommendations, and advice have been disseminated for years to practitioners in government and industry. The famous Seven Cardinal Rules of Risk Communication adopted by the U.S. Environmental Protection Agency (EPA) are an early example of academic risk communication findings being introduced to influence organizational practice (EPA, 1988). Such guidelines represent a response to problematic efforts to communicate risk by government agencies. Several ineffective endeavors have been reported in the literature. For example, risk communication conducted by the U.S. EPA in the case of a controversy over the remediation of contaminated land in Aspen, Colorado, although on article adhering to the seven cardinal rules of risk communication, nevertheless upon its practical implementation failed to build trust and overcome conflicts (Stratman, Boykin, Holmes, Laufer, & Breen, 1995). Other examples of communication failures, such as the alarm over acrylamide in food in Sweden (Löfstedt, 2008b; Renn, 2003), the aspartame scare in Europe (Löfstedt, 2008a), the Hallandsås railway tunnel crisis in Sweden (2007–2009) (Boholm, 2008, 2009), information to the public on climate change in Sweden (Uggla, 2008), and the communication in the United Kingdom of scientific findings that artificial food colorings can cause hyperactivity in children (Löfstedt, 2009), suggest that government agencies do not apply robust risk communication strategies.

So despite 40 years of academic study and abundant advice and recommendations the outcome is disappointing; there is little improvement in risk communication practices by government and industry (Kasperson, 2014). Unsurprisingly, as suggested by the above examples, there is a substantial gap between the recommendations derived from the study of risk communication and agency practice (Chess, Saville, Tamuz, & Greenberg, 1992). So, what explanations have been identified in the literature on why risk communication by organizations fails? Several studies point out that government agencies fail to address target groups by providing culturally relevant and meaningful information on risk; this is particularly valid in the case of local communities and minority groups (Chess, Burger, & McDermott, 2005; Driedger, Cooper, Jardine, Furgal, & Bartlett, 2013; Jardine, 2003; Jardine, Boyd, & Furgal, 2009). Another explanation put forward is that agencies lack shared perspectives (Chess, Salomone, & Hance, 1995; Chess, Salomone, Hance, & Saville, 1995). Government officials often have disparate ideas of public participation and may diverge in how they experience support and commitment within a given agency regarding communication with the public. Considerable variations in perspective on risk assessment and risk communication among officials with different roles in individual agencies have also been observed (Fisher, Chitose, & Gipson, 1994). Differences in how officials view risk assessment and risk communication do not seem to coincide with the particular agency, managerial position, or education of the official (Chess & Johnson, 2006). Although an understudied area in risk communication research (Chess & Johnson, 2010), findings so far on how risk communication is carried out in an organizational context suggest that there is considerable idiosyncratic variation in goals, priorities, working methods, means, and requirements.

This study answers to a need for more research on the organizational dimensions and dynamics of risk communication, which can answer questions such as how organizations operate, learn, and structure activities and make decisions (Chess & Johnson, 2010). By focusing in depth on the views of public officials at government agencies who work as risk communication practitioners, the study explores from a qualitative bottom‐up approach how risk communication is construed by the actors themselves. The objective of the study is to uncover what goals, principles, challenges, and *modus operandi* risk communication is supposed to include from a practitioner perspective. A further question is the practitioners’ familiarity with norms and principles of risk communication as developed within academia, and their thoughts on certain key topics discussed in the academic literature, such as the role of expert/scientific information, the role of uncertainty, and the role of transparency.

## 2. METHOD

The study is based on 23 interviews with public officials at six national agencies in the policy areas of food, chemicals, environmental protection, traffic infrastructure, contingency planning, and housing and zoning planning. Three agencies fall under the Ministry of Enterprise and Innovation, two under the Ministry of the Environment and Energy, and one under the Ministry of Justice. The selected agencies (age, size, responsibilities, location, and activities) are presented in more detail in Table I.

**Table I:** Selected Agencies with Background Characteristics

Agency	Location	Size	Government	Responsibility	Activity	Founded
The National Board of Housing, Building and Planning	Karlskrona and Stockholm	Ca 350	Ministry of Enterprise and Innovation	Societal planning, urban development, building, and accommodation	Regulation Collaboration	1988
The Swedish Transport Administration	Main office Borlänge and six regional offices	Ca 6 500	Ministry of Enterprise and Innovation	Road and railway traffic, sea traffic, and aviation traffic Maintenance and operation of state roads and railways Implement zero vision	Planning Operation of infrastructure Regulation (control) Procurement of collective transport	2010
The Swedish Environmental Protection Agency	Stockholm	Ca 500	Ministry of the Environment and Energy	Promote sustainable development regarding ecological dimensions, guided by environmental quality goals decided by the parliament	Implement and develop environmental policy, collaboration, regulation, research, and knowledge production, information, distribute economic funds	1967
Swedish Chemicals Agency	Stockholm	Ca 270	Ministry of the Environment and Energy	Law enforcement for chemical products Supervision and development of regulation	Inspection, guidance, information (databases, registers)	1986
National Food Agency, Sweden	Uppsala	Ca 530 (337 at head office)	Ministry of Enterprise and Innovation	Law enforcement for food and food products, supervision and development of regulation	Regulation, enforcement, recommendations, communication	1972
The Swedish Civil Contingencies Agency	Stockholm and Karlstad	Ca 850	Ministry of Justice	Civil protection, public safety, emergency management, and civil defense before, during, and after an emergency or crisis	Knowledge production, training, regulation, supervision, collaboration	2009

The sample originally included seven agencies but in the case of energy the organization decided not to participate in the study. The selected practitioners were all officials who work directly on communication and information at the agencies, that is, senior managers and managers as well as experts with specialist knowledge in specific fields. When the project started in 2015, the managing director of each selected agency was contacted and given information about the research project, its funding, and the research team. Each agency was asked to appoint a representative to a reference group attached to the project. These representatives all occupied high managerial positions in their organizations, having responsibility for communication. They were requested to provide names of practitioners to interview and were also themselves interviewed as well. The interviewee selection was therefore based on self‐selection by each organization according to criteria that were initially outlined, namely, that we wanted to interview practitioners who “worked on risk communication” and who could be expected to have experience and viewpoints relevant to the topic. All practitioners designated by their organizations consented to participate in the study and to have the interview recorded and transcribed. Anonymity was granted by not disclosing the name of the interviewee, or any other specific information regarding organizational position or agency affiliation in texts published on the material. Organizational dimensions of risk communication are not easily accessible (Chess & Johnson, 2010). Managers and practitioners might feel that researchers intrude and gain access to sensitive information in ways that might harm the organization's reputation and trust, and also create tensions within and between organizations.

The interviews were conducted between December 2015 and June 2016 by telephone using the speakerphone function and usually lasted around 90 minutes. The author conducted the interviews and a research assistant listened in, taking notes and sometimes asking additional questions. All interviews were recorded and transcribed verbatim. The interviews combined a semi‐structured approach with open‐ended conversations in which the interviewees were encouraged to speak freely, reflecting, free‐associating, and posing questions on the topics considered. The semi‐structured component meant that certain key general questions were introduced in the same way in all interviews. The open‐ended component was intended to capture each interviewee's understandings, thoughts, associations, and spontaneous responses to the topics introduced (Brinkmann, 2014; DiCicco‐Bloom & Crabtree, 2006).

Of the 23 interviewees, 12 were men and 11 women. With one exception four interviews were conducted at each agency. At the Chemicals Agency we did three interviews. The sampled practitioners consisted of communication managers and communication directors highly placed in the organizational hierarchy (six), senior managers heading a division or department (five), one press unit head, strategic advisors (four), communicators (three), and four others, that is, an expert, advisor, spokesperson, and coordinator. Regarding disciplinary background, six stated that they were social scientists (e.g., political scientists and sociologists), eight had backgrounds in the media, communications, and journalism, seven had backgrounds in natural science and engineering, and two had mixed backgrounds. The duration of employment at the agency varied considerably, ranging from 2 to 30 years: seven interviewees had worked over 10 years at the agency, and 10 had worked at the same agency between 5 and 10 years.

The interviews covered a number of areas: background questions (e.g., time in current position and educational background); work tasks; understanding of the agency's broader responsibilities; understanding of risk communication, the expert role, and scientific knowledge; views of uncertainty and transparency; and strategies and practices for communication (including relationships with the media and use of social media). In order to provide anonymity both for the interviewees and the individual agency, the agency source for quotes is not disclosed in the text.

## 3. RESULTS

The results are organized into six sub‐areas: ideas and understandings of the concept of risk and familiarity with and use of risk communication at the agency; ideas and norms about the expert/scientific component of risk communication; thoughts on how to address uncertainty; ideas and assumptions about the audience to which the communication is directed; ideas about the goals of communication (what does risk communication do and why is it needed); and thoughts about transparency and openness in risk communication.

### 3.1. The Concept of Risk and Familiarity with Risk Communication

The officials at the agencies varied greatly in their familiarity with the concept of risk. Only two interviewees actually ventured to define risk: “negative events that are possible” and “the probability and consequences of an event.” Only at the Food Agency did interviewees unanimously state that a risk framework was well established, even fundamental to their work. The European Food Safety Authority (EFSA) explicitly states that risk assessment is a central practice in agency work (EFSA, 2017a). EFSA has recently published an updated guideline and handbook on risk communication (EFSA, 2017b). For the other agencies there were no corresponding overall frameworks on risk assessment, management, or communication.

Among the 23 interviewees, five said that the concept was not used at their agency: risk was not discussed at all; risk was not a topic; there was no shared understanding of risk; and/or risk was not communicated. In seven cases when risk was addressed as a conceptual entity in one way or another, interviewees dwelled on the complications and difficulties of the concept. They said: “Risk deals with complicated matters”; “The concept may sound dramatic”; “It is a difficult concept, difficult to explain to people what risks are acceptable, difficult for the ordinary person to take a stance”; and “There is conflict between risk and hazard in European industry: those who think about economic profit want risk‐based [regulation]; some regulations are based on risk and there is a common view of how to conduct risk assessment, but the judgment of [what constitutes] high or low risk varies.”

Overall, two‐thirds of the interviewees did not address the concept of risk. Given the length of the interviews and the opportunities the interviewees were given to address the concept of risk, it is notable that so little was said about the meanings of risk and their implications. Apart from the Food Agency, officials at the Chemicals Agency were the only ones who dealt with risk as such. Officials at the Chemicals Agency often placed the “risk perspective” in relation, or rather in opposition, to the hazard approach predominant at their agency (Löfstedt, 2011).

We did not ask direct questions about whether or not risk communication was actually part of agency work. Because the interviewees had been selected by senior managers at their agencies to participate in the study, and because they knew beforehand that our research was about “risk communication,” we thought that answers to direct questions about whether or not they undertook risk communication would be biased. They might feel obliged to present a more idealized picture of familiarity with risk communication than was actually the case. We therefore probed the familiarity with and use of risk communication at the agencies more indirectly. Only four interviewees stated that they were familiar with risk communication or that risk communication was central to agency work. Those who expressed familiarity with the concept said: “We communicate risk every day”; “We work on risk communication all the time”; or “Risk communication is incredibly important.” However, seven interviewees spontaneously stated that they were unfamiliar with the term. They said that the approach was not used, and that there was no talk about risk communication at their agency; for example, “I am not used to the concept of risk communication, we don't use it” or even “We do not talk about risk communication at all, we have never talked about risk communication.”

Seven interviewees spontaneously compared risk communication with other types of communication, saying that other approaches were used instead, for example: “To be honest, I am not used to the concept [of risk communication]. We speak of crisis communication. I do not use risk communication myself. Either everyday communication or crisis communication.” These interviewees reflected on the differences and similarities between risk communication and crisis communication, some thinking that the approaches were fairly similar, while others thought they were different. Expressing a view shared by several, one interviewee said: “Risk communication and crisis communication are difficult to separate. It becomes difficult when something happens and there is a crisis. Crisis communication is not planned to the same extent as is risk communication.” Another risk practitioner focused on risk communication as part of risk management: “I would like to propose that to work with society and on contingency preparedness and to prevent and manage [risks], we must communicate risks and threats and have tools for systematically identifying risks and threats. For systematically evaluating whether you want to live with risks or whether they should be fixed.” This interviewee understood risk communication largely in a classical way as an element of risk analysis associated with risk management.

### 3.2. The Expert/Scientific Component of Risk Communication

Overall, the interviewees strongly believed in science and expert knowledge as the foundation of risk communication. More than two‐thirds (14) of the interviewees stressed that scientific and expert knowledge was important, very important, or extremely important for risk communication at the agency, saying things such as: “It is important in providing substance for facts”; “[It is] extremely important, we could not do without it”; and “[It provides] quality based on knowledge and competence—this is alfa and omega.” Why did the interviewees attribute such a profound role to science and expert knowledge in risk communication?

A first observation is that most interviewees seemed to understand science as a source of fundamental noncontestable value. The interviewees strongly believed that risk communication should be based on science. Science was understood as “objective,” consisting of “solid bodies of facts” and “scientific methods,” which together provide “profound knowledge of a specific area.” Statistical knowledge was also mentioned in relation to science, for example, in that science reveals “what the statistics say.”

Scientifically based information was furthermore assumed to have specific effects on the receiver. It could exert “influence,” create “calm,” and “provide information without scaring or trivializing.” It was also understood to give information weight and legitimacy. Eleven interviewees stressed trust in science as a key requirement of risk communication, emphasizing that “we trust experts.” For example, one stated that “a traffic expert who talks about weather and slippery roads has greater credibility.” Science and expert knowledge were understood to “contribute to openness, creating trust.” The presence of experts as communicators was understood to promote trust, “giving the impression that the agency has knowledge” and “creating credibility … people trust us [because] science is independent, [has] no political interests, and creates openness—our entire credibility is built on independent science.”

However, the practitioners also thought that a strong science component and over‐reliance on experts in risk communication had a downside. Scientific experts could for various reasons also be obstacles to effective risk communication. First, it was recognized that experts are not always the best communicators with the public (six interviewees). The information they provide might be too theoretical and too complicated, and therefore not easily understood by nonexperts. Some interviewees said that experts “have an over‐reliance on information,” and that they “do not address citizens.” These interviewees therefore thought that “science must be reformulated so that it can be communicated.” A second problem noted was scientific controversy or disagreement. Six interviewees mentioned that disagreement between experts or between conflicting scientific opinions could be a problem. They thought that conflicting views of a risk topic might derive from different scopes of knowledge, some experts having narrower and others broader knowledge. A particular challenge identified was how to decide between and balance different types of knowledge and conflicting viewpoints in risk communication. A general experience was that such differences can be difficult to communicate, presenting a substantial challenge.

### 3.3. How to Address Uncertainty

Another topic addressed was how to communicate uncertainty in relation to risk. The interview guide contained a question about how uncertainty about risk issues was dealt with in communication at the agency. Because several respondents seemed to have rather vague understandings of the concept of risk or were unfamiliar with its meanings and uses, we did not really press them on the matter of uncertainty. We did not ask them to define uncertainty or to consider the concept in greater depth. Nevertheless, we did get some input, either in response to direct questions or offered spontaneously by interviewees during the conversation.

One issue raised by eight interviewees concerned uncertainty in risk assessment, that is, epistemological uncertainty about how to factually characterize risk in a certain context. For example, one interviewee emphasized: “There are three uncertainties: Is there a [factual] basis—is something carcinogenic or not? Scientific uncertainty—how can we draw conclusions? And statistical uncertainty?” Another issue that came up is lack of knowledge of how human behavior and societal conditions influence risk. For example, there might be a lack of knowledge about exposure to harmful substances (“How do people actually use chemicals?”) and a lack of knowledge about “overall trends and developments in society,” which might be crucial to how to assess risks.

Six interviewees emphasized that it was important as a general principle to communicate uncertainty. They thought that their agencies should present all the various aspects of a risk issue, conveying “what we know and what we do not know,” for example, using the word “may” (Swedish, *kan*) to signal that something may have negative effects, and to convey that science knowledge is open to revision.

Some interviewees did not share this belief in the notion of openness. They were instead skeptical about openly admitting to uncertainty in risk assessment and risk management. For example, one interviewee explicitly argued that risk communication must convey clear messages about risk management measures, that is, clear instructions as to how a risk might be mitigated, otherwise the agency might lose public trust:

If one says “Watch out!”—but for what? The risk must be reasonably well defined. There must be something to communicate. This is about our trustworthiness, [our] expertise. Should we communicate a risk that we ourselves have hardly kept track of?

Another issue in relation to uncertainty raised by five interviewees concerned disagreement between experts or between conflicting bodies of knowledge. Sometimes, such disagreement was internal to the agency, which one interviewee found difficult to handle: “Sometimes there is in‐house disagreement, on one hand, on the other … [it is] not easy to understand at all times.” In other cases, disagreement was identified between different types of experts or between experts and external stakeholders. The interviewees did not refer to any agency guidelines or strategies for dealing with uncertainty in risk communication, whether deriving from lack of knowledge of risk issues, scientific conflicts, or inconclusive evidence, or from lack of clear and practically feasible risk management actions.^1^

### 3.4. The Audience of Risk Communication

Twenty interviewees, a clear majority, thought that the audience addressed by risk communication comprised members of the public understood as individuals. Expressions used were “individuals,” “people,” “private persons,” “citizens,” and “consumers.” However, three interviewees explicitly ruled out members of the public as the audience of risk communication, stating: “It is not part of our mission to communicate to citizens”; “We seldom have dialogue with ordinary consumers, [though] occasionally someone calls”; and “We do not communicate directly with the public—other authorities are closer to the public.” Apart from the public, other addressees mentioned were “third parties” (mentioned by two interviewees), the European Commission (mentioned by one), municipalities (mentioned by three), regional county boards (mentioned by two), politicians and decisionmakers (mentioned by one), and practitioners, branch representatives, and organizations (mentioned by two). Clearly, members of the general public in their roles as private persons, citizens, and consumers were generally assumed to be the main audience of risk communication. Overall, interviewees envisaged communication with individual members of the public as one‐way communication. The agencies themselves were not thought of as the recipients of information, except for information from experts. Very little in the interviews indicates any two‐way communication with the public.

### 3.5. The Goals of Risk Communication

Regarding the goals and objectives of risk communication as understood by the practitioners, six main themes can be identified:

(1) Educational: disseminate knowledge, explain, create awareness, influence attitudes, and make people listen (mentioned by 22 interviewees).
(2) Behavioral: influence action and decision making (mentioned by 13).
(3) Contingency management: manage, avoid, minimize, or eliminate risk (mentioned by 11).
(4) Psychological: manage emotions (mentioned by nine).
(5) Administrative/organizational: fulfill organizational goals and follow procedures for decision making (mentioned by six).
(6) Reputational: promote legitimacy and trust (mentioned by five).

These goals are not exclusive, and individual interviewees often touched on several goals in their interviews.

The most common category of stated goals is *educational*, mentioned by almost all interviewees (22). In this category we find aims such as disseminating knowledge of risk, providing information on risk, heightening awareness of risk, and creating interest in and understanding of risk. Sometimes, the educational goal was further qualified by statements that the information must be objective, factually correct, balanced, and easily understood. A shared assumption is apparently that risk communication entails the pedagogical presentation of easily understood factual knowledge of risk to the public.

The second most common category of goals, mentioned by more than half of the interviewees (13), is *behavioral*. Here we find the aims of influencing behavior, action, and decision making. This category of goals reflects a classical interest in the risk communication literature deriving from its close affinity to decision theory (Arvai, Gregory, & McDaniels, 2001). It should be noted that there is a division regarding the interviewees’ assumptions about the relationship between risk communication, on one hand, and decision making versus behavioral change, on the other. One group of answers suggests that the goal of risk communication is to provide information to individuals so that they themselves can make up their minds and make decisions. Interviewees stated, for example, that risk communication helps people “to make their own decisions” and “make free choices.” Another type of answer stated that risk communication was designed by the agency with the specific aim of influencing the outcome of individuals’ decisions and actions, for example, to “change behavior to become more climate friendly” or to “make people act so that their health improves.”

These two types of answers suggest that perspectives diverged between (and within) agencies as to the capacities of the addressee. A more liberal position delegates the decisions to the individual, understood to have the capacity to make the best decision providing the appropriate information is available. However, from a more technocratic top‐down perspective, the individual is not seen as capable of deciding what the best action is, but rather needs to be guided in a certain direction, primed by information from the agency. This illustrates a classic contrast in risk communication between persuasive and merely responsible government (Leiss, 1996).

The third most common category of risk communication goals is *contingency management*, that is, to manage, avoid, minimize, or eliminate risk (mentioned by 11 interviewees). For example, interviewees stated that risk communication is instrumental in “avoiding risk,” “preventing accidents, being proactive,” and “serving taxpayers—as a car driver you should not be surprised when there is road work and queuing.” Communication about risk creates awareness, which is central to effective risk management. This category offers an instrumental perspective according to which risk communication is understood to provide results in terms of improved safety.

The fourth most common category of goals mentioned by more than a third (nine) of the interviewees refers to *psychological* aims. This goal relates to the management of emotions, mainly negative emotions such as fear and anxiety. Here, risk communication is understood as a way to reduce fear among the public by providing factual information about potential negative events in a way that does not evoke undesired negative emotions. As interviewees noted, risk communication makes “people aware without frightening them” so they “understand and are not unnecessarily worried”; moreover, risk communication allows people to “manage that which evokes worry.” The underlying rationale is that “people should feel calm.”

This category of aims suggests that agency officials assume that the general public is emotional and not entirely rational. Members of the public respond to risk with unnecessary fear and worry, and risk communication can steer these negative emotions to establish a more neutral emotional state, namely, “feeling calm.” Only one interviewee directly questioned this psychological aim of risk communication, stating that it is *not* the goal of risk communication to make people calm. On the contrary, this interviewee argued that there are indeed many risks that members of the public should take more seriously and strive to address: sometimes the public ought to be more worried, not less.

One category of risk communication goals relates to internal and organizational dimensions. The theme of *administrative/organizational* goals places risk communication in a broader organizational setting and policy context (mentioned by six interviewees). In this theme, risk communication is understood as an administrative requirement covered by decision‐making procedures within the agency. It is something that the agency is required to do and it fulfills internal demands such as to “create a good basis for decisions” and “meet operational goals.” Another category of risk communication goals concerns *reputational* issues (mentioned by five interviewees). The interviewees who addressed this theme all assumed that risk communication creates or affects public trust. Two interviewees also related risk communication to the “brand” of the agency: risk communication is believed to “protect trust and the brand,” helping maintain the agency's public image, or brand.

### 3.6. Transparency and Openness in Risk Communication

In some cases, transparency was mentioned spontaneously by the interviewees at the beginning of the interview when they were talking about their work and their agencies’ responsibilities, when they were characterizing and reflecting on risk communication. We also asked some specific questions about transparency toward the end of the interview. The interviewer opened with a statement: “Risk communication and transparency is a tricky area—openness can be understood to be both good and bad, it depends … .” The interviewer then asked whether openness was discussed in the organization, whether there were policies about what to communicate, and why, and finally whether openness was good or bad for risk communication on the whole.

A clear majority of 17 interviewees stressed that openness was important or very important in their organization. For example, they stated: “We discuss it, it is the only way”; “It is very important—we are more open than other agencies”; “Openness is a key word—we do not struggle to balance openness”; “In general we believe in transparency regardless of the situation”; and “We think about openness all the time—it must be easy to find things out and [information] must be easy to understand.” Only one interviewee stated that openness or transparency was not discussed in the organization. Three interviewees had a moderate belief in the importance of transparency, saying, for example, that “as it happens, the topic does not come up so often” or “we say that we want to be open, that we want openness.” In the latter statement, openness as an ideal is understood to work differently in practice. Four interviewees explicitly connected openness and transparency to risk communication. The key elements emphasized were: (i) not hiding information and (ii) that information should be available; for example, “[it is] fundamental to risk communication not to hide”; “[it is] central to risk communication that material be available all the time.”

Ten interviewees in various ways indicated what they believed constituted the goals of transparency. The most often mentioned goal was that of building trust in and the credibility of the agency (mentioned by four interviewees), followed by providing information as a service to citizens and stakeholders, including public access to documents according to the constitution (mentioned by four), fostering accountability by disclosing how the agency works and makes decisions (mentioned by two), and, finally, creating dialogue (mentioned by only one).

Transparency and openness in risk communication were also understood to be difficult to achieve. Sixteen out of 23 interviewees identified various challenges, problems, and obstacles encountered in striving for transparency. One topic related to a goal of risk communication referred to above, namely, managing the emotions of the addressees (i.e., members of the general public or specific target groups). Five interviewees said that it was difficult to communicate risk in an open way without creating fear; for example, one interviewee said: “We want to be open but we do not want to scare people.” Another issue mentioned was that it was difficult to translate expert knowledge into information that people could understand (mentioned by four interviewees); for example, one interviewee said that “people have difficulties understanding risk, they draw the wrong conclusions. We have to explain [it] in the right way—some things cannot be communicated.”

In connection with challenges identified in implementing openness in risk communication, several interviewees emphasized difficulties in deciding what to present and what not to present, talking about balancing information about risk in practice. One interviewee mentioned differences in transparency “culture” between agencies as a problem: “Different agencies have different views of transparency. If the expert perspective is strong, transparency might be difficult, and agencies that have a gut feeling about nontransparency can be difficult to co‐operate with.” Conflicting goals between informing the public and not creating adverse side effects were also identified. One interviewee cited an example of this: transparency in risk communication “can affect companies—the bean sprout incident hit an entire industry and sales were halved.”^2^

One particular aspect of openness in risk communication is security. Six interviewees specifically talked about goal conflicts between transparency and security, mentioning cases when making information public might pose a threat to national security, agency staff, or protected objects in the sectoral policy area of agency responsibility. For example, one interviewee mentioned that if the agency provided public information on the locations of king eagles’ nests, this could threaten the eagles by enabling visits by people who for various reasons might disturb or harm them. Two interviewees noted the many digital files and records that could be used by antagonistic actors. One interviewee said: “We are too naïve and open, as there are possible terror scenarios in which Swedish infrastructure could be a target. A more emphatic discussion is needed. There are switchgear stations, railway signal systems, and bridge structures.” Another interviewee had similar concerns, stating that “we should not reveal vulnerabilities so that they can be used in the wrong way.”

On one hand, transparency as openness in information provision is applauded as a noble principle of government; on the other, transparency in practice is understood as difficult to implement due to several identified adverse effects, such as security threats and causing possibly unjustified fear among members of the public. The risk communication practitioners studied here understood transparency as good and necessary, and as simultaneously difficult to achieve in practice due to the many complex decision situations that arise.

## 4. DISCUSSION

In the Swedish regulatory climate, collective “elite stakeholders” (e.g., trade unions, industry, and interest organizations) have a long tradition of being privileged participants in the policy process. Courts of law have a limited role in regulatory policy making (Löfstedt, 2005). Corporatism, collaboration, and consensus among state officials, regulators, the political elite, and collective elite stakeholders are distinctive features of policy and regulation (Löfstedt, 2005). However, there are indications that corporatism declined in the late 1980s and early 1990s, at least in some policy areas (Lindvall & Sebring, 2005). Public trust in institutions (e.g., politics, corporations, and government bodies) is higher in Sweden than in other European countries (Hudson, 2006; Viklund, 2003). There is a strong technocratic tradition of risk regulation, which is overall conflict averse. Expert advice has a strong role in public policy and the role of citizen deliberation and participation is limited (Löfstedt, 2005).

A first observation from the interview material is that “risk,” except at the Food Agency, was not advanced as an organizing principle of communication practices. At some agencies, practitioners even explicitly said that a risk perspective was absent. This result contradicts the common assumption in the literature that a risk framing has “colonized” regulation (Rothstein, Huber, & Gaskell, 2006) or that nowadays there is the risk management of “everything,” in which risk and its regulation, management, and communication are increasingly invoked in private and public organizations at a global scale (Power, 2004, 2007).

Another argument in the literature is that a new style of risk regulation has emerged in Europe where, due to decreasing public trust, agencies have responded by enhancing public participation as a regulatory measure (Löfstedt, Bouder, Wardman, & Chakraborty, 2011). Contrary to such assumptions, we found that the studied practitioners largely adhered to a traditional, technocratic, top‐down model of risk communication. Dialogue with the public was said to be virtually nonexistent, communication with the public or affected groups being predominantly one‐way. Dialogue and participation were, however, practiced with other organizations, elite stakeholders, and other public administrations, but not with the public. The assumption of rapidly expanding risk awareness in public affairs, government, and business is not substantiated by our results. Neither are assumptions about an increased role of public participation and transparency in risk regulation. One explanation for the low saliency of a risk framing in policy and regulation might be the relatively high public trust in government institutions that characterizes Swedish society.

Despite a long tradition of academic critique of scientific knowledge as a foundation for risk communication, scientific and expert knowledge emerged as key to the practice of risk communication among the agencies studied. Apart from problems and uncertainties raised by scientific disagreement and controversy, there was very little critical reflection among the practitioners on the role of science in risk communication. Very little of the academic discussion of how to make risk communication more democratic and responsible seemed to have reached the level of practice (Leiss, 1996; Power, 2004). The role of science continued to be strong and experts retained a privileged position, although admitted problems with an excessive reliance on science made it difficult to communicate comprehensible messages to the public. The interviewees reported little or no effort to explore the actual concerns of members of the public. In line with the traditional, technocratic paradigm of risk communication, members of the public tended to be construed as irrational, governed by emotions and responding to risk with fear or anxiety (Leiss, 1996; Wardman, 2008). No shared paradigm of risk communication was evident among the agencies. In fact, knowledge of the academic study of risk communication and its findings and recommendations was, with one exception, minimal or nonexistent at the agencies.

A common idea among the practitioners was that a main goal of risk communication is to provide information in a way that does not promote fear. Compared with the traditional, technocratic, top‐down model, there was much less emphasis on persuasion among the practitioners (Leiss, 1996). Although some did think that persuasion, to induce people to accept certain risk framings and certain risk management measures, was a goal of risk communication, most practitioners expressed the more “liberal” idea that by being informed about risk, members of the public could make their own decisions. This is a more enlightened vision of risk communication in which the state, through public authorities, provides relevant information on risk but without taking a side as to what decisions should be made (Arvai, 2014; McComas, Arvai, & Besley, 2009).

The perceived goals of risk communication varied greatly among and within the agencies. The practitioners had more or less shared and idiosyncratic understandings of what risk communication entails, of what it does, and of why they should do it, making risk communication elusive and difficult to evaluate. The expectation of Leiss in the 1990s regarding the third phase of risk communication (1996–present), that a “code of good risk communication practice” together with a framework for “risk communication audit” would be in place for evaluating and testing public outreach, has not yet been realized (Leiss, 1996, p. 94).

Risk communication was also understood by the practitioners as having trust‐promoting functions and the potential to affect organizational reputation, strengthening the organization's “brand” if carried out properly (or potentially the opposite if executed badly). These goals have been discussed extensively in the academic literature on risk communication, when failures of the classical paradigm have been identified (Power, 2004, 2007; Rothstein et al., 2006).

Transparency is a broad and ambiguous term with both descriptive and normative implications that potentially harbor conflicts between goals (Bannister & Connolly, 2011; Hood & Heald, 2006). Openness or transparency in risk communication, seen in the academic literature as a key norm, was recognized by most risk practitioners as crucial at least in principle. However, when the interviewees reflected on the matter more practically, a number of conflicts, problems, and tensions were identified: disclosed information might pose a threat to security if it is used by antagonistic agents; information signaling high uncertainty might have negative side effects, promoting fear among the public and potentially loss of trust in the agency; and there are tensions between avoiding blame and disclosing information (Hood, 2007). It is clear from the practitioners’ views that transparency in risk communication is not an easy remedy for risk regulation problems relating to trust and legitimacy (Bouder, Way, Löfstedt, & Evensen, 2015; Lofstedt, Bouder, & Chakraborty, 2013; Way, Bouder, Löfstedt, & Evensen, 2016).

## 5. CONCLUSIONS

There is a substantial gap between the academic study of risk communication and the advocated requirements for risk communication, on one hand, and government agency practice, on the other. This is disappointing considering the massive efforts of risk communication scholars to explore, explain, criticize, evaluate, and make recommendations with the aim of improving practice. One immediate implication for better risk communication practice at the agency level is that education and training, specifically in academic understandings of risk communication, need to be prioritized at the agency level. The agencies have already devoted resources to communication and information skills in general, but this is not enough. Specific attention needs to be paid to what risk analysis means, and to the goals, practices, and procedures that this framework encompasses. Furthermore, government agencies need to exchange learning, views, and perspectives regarding key issues across agencies, such as the communication of uncertainty and how to work with transparency. Perspectives on risk and risk communication, its goals, methods, and outcomes, need to be harmonized. More research into agencies in other countries, inside and outside the European Union, is greatly needed. National differences with regard to risk communication practices and practitioners’ understandings and experiences are clearly to be expected (Rothstein et al., 2017). For academic risk communication to have an impact on practice, we need qualitative knowledge of how risk communication work is actually carried out and understood by the practitioners themselves in contexts of national government agencies. Further research also needs to address how the implementation of other policy goals and objectives influences organizational risk communication work at government agencies.

## Footnotes

1 The updated EFSA guidelines and handbook on risk communication referred to above specifically address uncertainty included under the principle of transparency (p. 11).

2 The interviewee is referring to a food scare in 2011 when bean sprouts produced in Germany were found to be contaminated with Enterohaemorrhagic Escherichia Coli (EHEC). In Sweden, the sales of all varieties of sprouts were stopped for a period.
